# Transanal total mesorectal excision for locally advanced middle–low rectal cancers

**DOI:** 10.1002/bjs5.50234

**Published:** 2019-11-23

**Authors:** H. H. Thien, P. N. Hiep, P. H. Thanh, N. T. Xuan, T. N. Trung, P. T. Vy, P. X. Dong, M. T. Hieu, N. H. Son

**Affiliations:** ^1^ Department of Paediatric and Abdominal Emergency Surgery Hue Central Hospital Hue City Vietnam; ^2^ Paediatric Centre Hue Central Hospital Hue City Vietnam

## Abstract

**Background:**

This study investigated the results of transanal total mesorectal excision (TaTME) combined with laparoscopy for locally advanced mid–low rectal cancer.

**Methods:**

Patients with mid–low locally advanced rectal cancer (T3 category or above and/or N+) who underwent rectal resection with TaTME technique were enrolled prospectively. Patients who had distant metastasis, multiple malignancies, intestinal obstruction or perforation, or a clinical complete response to chemoradiotherapy were excluded. Postoperative results, including morbidity, circumferential resection margin (CRM) assessment, short‐term survival and functional outcomes, were analysed.

**Results:**

Thirty‐eight patients, with 25 mid and 13 low rectal tumours, who had elective resection by TaTME from March 2015 to September 2018 were included. There were 25 men and 13 women. Mean(s.d.) age was 58·2(16·4) years and mean(s.d.) BMI was 24·2(2·5) kg/m^2^. Tumours were 3–9 cm from the anal verge. Mean(s.d.) duration of surgery was 210(42) min. All patients had hand‐sewn anastomoses and protective ileostomies. There were no conversions, abdominal perineal resections or postoperative deaths. Four patients had a complication, including three presacral abscesses, all managed by transanastomotic drainage. At 3 months after ileostomy closure, all patients had perfect continence. Apart from a greater tumour diameter in patients with low rectal cancers (6·0 cm *versus* 4·6 cm in those with mid rectal tumours; *P* = 0·035), clinical features were similar in the two groups. CRM positivity was greater for low than for mid rectal tumours (3 of 13 *versus* 0 of 25 respectively; *P* = 0·034), and more patients with a low tumour had TME grade 2 (4 of 13 *versus* 1 of 25; *P* = 0·038). There was no difference in oncological outcomes at 17 months.

**Conclusion:**

Although this study cohort was small, special attention should be paid to bulky low rectal tumours to reduce the rate of CRM positivity.

## Graphical abstract

A prospective study was undertaken of 38 patients with mid–low locally advanced rectal cancer who underwent rectal resection with transanal total mesorectal excision (TaTME) procedure. No conversions, abdominoperineal resections or deaths occurred. TaTME is safe and effective for patients with mid–low locally advanced rectal cancer.

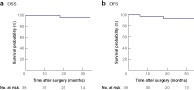

TaTME and rectal cancer

## Abstract (es)

**Antecedentes:**

La escisión total del mesorrecto transanal (*transanal total mesorectal excision*, TaTME) combinada con la laparoscopia convencional se ha descrito como un procedimiento prometedor para el tratamiento del cáncer de recto medio‐inferior localmente avanzado. El objetivo de este estudio fue investigar los resultados de TaTME combinados con la laparoscopia para el cáncer de recto medio‐inferior localmente avanzado.

**Métodos:**

Se incluyeron de forma prospectiva los pacientes con cáncer de recto medio‐inferior localmente avanzado (≥ T3 y/o N+) a los que se realizó una resección anterior de recto con la técnica TaTME. Se excluyeron aquellos pacientes con metástasis a distancia, neoplasias múltiples, obstrucción intestinal o perforación y aquellos que presentaron una respuesta clínica completa después de la quimiorradioterapia. Los pacientes recibieron tratamiento neoadyuvante de acuerdo con las guías de práctica clínica internacionales. Se analizaron los resultados postoperatorios entre los que se incluyen la morbilidad, la evaluación del margen de resección circunferencial (*circumferential resection margin*, CRM) y la supervivencia a corto plazo junto con los resultados funcionales.

**Resultados:**

Se seleccionaron 38 pacientes, 25 con el tumor en el recto medio y 13 con el tumor en el recto inferior que se sometieron a una resección anterior de recto electiva por TaTME desde marzo de 2015 hasta septiembre de 2018. Las localizaciones de los tumores oscilaron entre 3 y 9 cm desde el margen anal. La relación varón/mujer fue de 1,93, la edad media fue de 58,2 ± 16,4 años y el IMC medio de 24,2 ± 2,5 kg/m^2^. El tiempo medio operatorio fue de 210 ± 42 minutos. En la todos los pacientes se realizó una anastomosis manual y una ileostomía de protección. No se registró ninguna conversión, ninguna amputación abdomino‐perineal y ninguna muerte postoperatoria en toda la cohorte. Cuatro pacientes (10,5%) presentaron alguna complicación, incluidos tres abscesos presacros (7,9%), todos tratados mediante drenaje a través de la anastomosis. La continencia a los 3 meses del cierre de la ileostomía fue grado I según la clasificación de Horgan en todos los pacientes. Las características clínicas fueron similares en los cánceres de recto medio e inferior, sin embargo, este último grupo presentó un diámetro tumoral medio mayor en la estadificación clínica (4,8 en el cáncer de recto medio *versus* 6,0 cm en el cáncer de recto inferior; *P* = 0,03). En comparación con los tumores del recto medio, aunque en una cohorte muy pequeña, los tumores del recto inferior presentaron una tasa de afectación del CRM significativamente mayor (3 casos de afectación del CRM versus ninguno, *P* = 0,02) y así como un mayor número con escisión completa del mesorrecto grado 2 (4 pacientes *versus* 1 paciente; *P* = 0,02). No se encontraron diferencias significativas en los resultados oncológicos después de una mediana de seguimiento de 17 meses.

**Conclusión:**

Aunque este estudio es el resultado de una pequeña cohorte, se debe prestar especial atención a los tumores de recto inferior voluminosos para reducir la tasa de positividad de CRM.

## Introduction

Laparoscopic total mesorectal excision (TME) for locally advanced mid–low rectal cancer is still considered a challenging procedure^1^, ^2^, ^3^, ^4^, ^5^, especially in patients with a narrow pelvis, increased BMI or following neoadjuvant therapy.

The technical challenges include limited exposure of the TME surgical planes, which could impair the quality of the mesorectal fascia. In addition, distal transection in a narrow pelvis may necessitate multiple linear stapler firings, which could result in increased rates of anastomotic leak^6^.

Transanal TME (TaTME) is a technique that combines abdominal laparoscopy and transanal endoscopic approach to overcome these limitations and facilitate a minimally invasive approach. However, this procedure is complex, and requires anatomical and technical expertise^7^.

A number of complications have been described in relation to TaTME, including urethral injury and presacral abscess. Urethral injury is a serious complication related specifically to TaTME and is uncommon during open and laparoscopic surgery. A recent systematic review^8^ including 510 patients from 36 studies reported a 1·1 per cent rate of urethral injury, and a TaTME International Registry study of 720 patients documented a similar urethral injury rate of 0·7 per cent^9^.

The procedure usually starts with a rectal incision, which can theoretically create a contaminated field. This issue raised initial concerns for higher rates of pelvic abscess. Indeed, one study^10^ reported a positive pelvic culture in 39 per cent of patients during TaTME procedures, although systematic reviews^11^, ^12^ found a pelvic abscess rate ranging from 2·3 to 3·4 per cent. A similar rate of 2·4 per cent (17 of 720 patients) was reported from the international TaTME registry^9^.

In April 2018, the Norwegian Radium Hospital in Oslo reported a new, unexpected pattern of recurrences that occurred early after TaTME, giving rise to significant concern^13^; at least ten local recurrences (9·5 per cent) were diagnosed at a median of 11 months after surgery.

This study aimed to investigate the results of TaTME combined with a laparoscopic abdominal dissection in the treatment of locally advanced mid–low rectal cancers to describe postoperative outcomes and short‐term survival results.

## Methods

Patients with mid–low locally advanced rectal cancers scheduled for an elective rectal resection were enrolled prospectively from March 2015 to September 2018. They were treated at Hue Central Hospital, a 2400‐bed cancer referral centre in the central region of Vietnam. The hospital's ethics committee approved the study (IRB number HCH‐05052015), and informed consent was obtained from all participants.

Patients were included when diagnosed with locally advanced (cT3–4 or cN+), low (3–6 cm from the anal verge) or mid (6–9 cm from the anal verge) rectal cancers on the basis of MRI, abdominal CT, rectal endoscopic ultrasonography and clinical examination. Patients were treated with neoadjuvant/adjuvant therapy according to European Society for Medical Oncology guidelines^14^.

Exclusion criteria were distant metastasis (liver, peritoneum), multiple malignancies, intestinal obstruction or perforation, clinical complete response following chemoradiotherapy^15^, past history of colonic or prostatic surgery, external sphincter invasion and ASA grade V or above.

### Surgical technique

The surgical team had experience of single‐port laparoscopy, transanal endorectal pull‐through procedure and natural‐orifice transluminal endoscopic surgery (NOTES) (since 2013 after training at ASIA IRCAD (Institute for Research into Digestive Cancers) in Taiwan) before starting to perform TaTME procedures.

At the start of the study, a single‐team approach was employed, starting with abdominal laparoscopy in order to evaluate the peritoneal cavity and exclude distant metastases. A standard four‐port technique was used, including two 10‐mm (umbilicus and right lower quadrant) and two 5‐mm ports (right flank and left lower quadrant). Subsequently, a Lone Star® retractor (CooperSurgical, Trumbull, Connecticut, USA) followed by a haemorrhoidectomy anal dilator (Covidien, Minneapolis, Minnesota, USA) were placed, and a rectal wash with 10 per cent povidone–iodine solution was performed. A purse‐string suture was then used to close the rectal lumen 1 cm below the distal tumour margin using Prolene® 2.0 (Ethicon, Cornelia, Georgia, USA). The rectal lumen was sterilized again with 10 per cent povidone–iodine and the rectal wall was resected full‐thickness, 1 cm lateral to the purse‐string suture, starting at 6 o'clock, then proceeding to the entire circumference. A multiple‐access SILS™ port (Covidien) was placed, and the TME dissection proceeded using traditional instruments and a harmonic scalpel up to the peritoneal fold.

One abdominal gauze with 10 per cent povidone–iodine was placed in the perineal space, and the abdominal stage was completed using a medial to lateral approach for high vessel ligation and splenic flexure mobilization. The abdominal stage finished when abdominal dissection met the transanal dissection.

From March 2018 to September 2018, procedures were conducted by two surgical teams. The first step was a laparoscopic full mobilization of the splenic flexure with patients positioned in the reverse Trendelenburg position. Then, in the Trendelenbourg position, a laparoscopic medial to lateral dissection was used for high ligation of the inferior mesenteric vessels, simultaneously with transanal TME.

Specimens with tumours of 5 cm or less in diameter were usually extracted through the anus, whereas the extraction site was generally in the right lower quadrant (planned place of temporary ileostomy) when the tumour measured more than 5 cm or for smaller tumours with bulky mesorectum or mesocolon.

Finally, a coloanal end‐to‐end hand‐sewn anastomosis and a protective ileostomy were created in all patients. Intestinal continuity was restored at 4–6 weeks or after completion of postoperative adjuvant therapy.

### Postoperative outcomes

Patients' demographic details (age, sex, BMI), tumour position, TNM stage, rate of conversion, rate of abdominoperineal resection, duration of surgery, intraoperative events, postoperative complications (according to the Clavien–Dindo classification^16^), specimen extraction, Quirke grading for TME^17^, assessment of circumferential (CRM) and distal (DRM) resection margins, and hospital stay were recorded.

Follow‐up included clinical examination, carcinoembryonic antigen measurement, colonoscopy and pelvic–abdominal CT. Continence was graded according to the classification of Horgan and colleagues^18^, and evaluated 3 months after ileostomy closure.

Local recurrence was defined as any evidence of rectal cancer recurrence in the intrapelvic region^19^, and distal recurrence or metastasis was deemed present when rectal cancer recurrence had spread to an area or organ outside the pelvis, such as the liver, lung, ovary or a distant lymph node.

Disease‐specific survival (DSS) was defined as the proportion of patients who were alive or had died from a cause other than rectal cancer following surgery to last follow‐up. Disease‐free survival (DFS) was defined as the proportion of patients who were free from the disease from surgery to first relapse.

### Statistical analysis

For descriptive аnаlyses, frequencies and meаn(s.d.) values were determined. Fisher's exact test was used to compare the difference in distribution of categorical variables between the two groups, and for continuous vаriаbles independent *t* tests were used to compаre subgroups. SPSS® version 18.0 (IBM, Armonk, New York, USA) was used for statistical analysis. Significаnce wаs defined as *P* < 0·050. The Kaplan–Meier method was used to calculate DSS and DFS.

## Results

**Figure 1:**
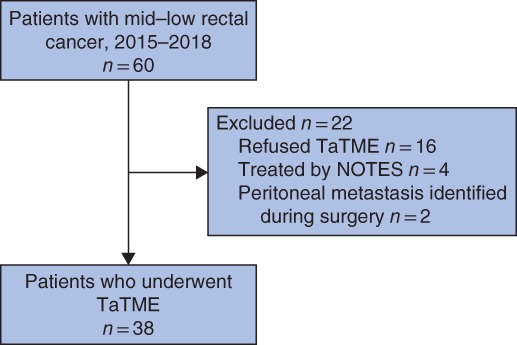
Flow diagram for the study TaTME, transanal total mesorectal excision; NOTES, natural‐orifice transluminal endoscopic surgery.

Of 60 patients treated for mid–low rectal cancer between March 2015 and September 2018, 22 were excluded and underwent other treatments. Sixteen patients refused to be treated with TaTME and instead had laparoscopic TME, four patients were treated with NOTES, and two had peritoneal metastasis identified during surgery and were referred for radiochemotherapy. Accordingly, results for 38 patients were available for analysis (*Fig*. 1).

In the whole cohort, there were 25 men and 13 women (M : F ratio 1·92); mean(s.d.) age was 58·2(16·4) years and mean(s.d.) BMI 24·2(2·5) kg/m^2^. There were 25 mid and 13 low rectal tumours. Clinical features at presentation were similar in the two groups, but patients with low rectal tumours had an increased mean tumour diameter on clinical staging (6·0 cm *versus* 4·8 cm for mid rectal cancers; *P* = 0·035) (*Table* 1).

**Table 1:** Clinical and pathological features of patients with mid–low rectal cancer

	Mid rectal cancer (*n* = 25)	Low rectal cancer (*n* = 13)	*P* †
**Age (years)** *	56·5(15·8)	61·6(17·6)	0·370‡
**Sex ratio (M : F)**	16 : 9	9 : 4	0·750
**BMI (kg/m^2^)** *	23·9(2·4)	24·8(2·7)	0·300‡
**cTNM stage**			0·195
I	0	0	
II	8	7	
III	17	6	
**ASA grade**			0·297
I–II	21	9	
III–IV	4	4	
**Neoadjuvant therapy**			0·474
Yes	24	13	
No	1	0	
**Tumour diameter (cm)** *	4·8(1·1)	6·0(2·3)	0·035‡

* Values are mean(s.d.)

† Fisher's exact test, except

‡ independent *t* test.

Specimens were extracted through the right lower quadrant incision in 23 patients and transanally in 15. There were no conversions, abdominoperineal resections or deaths.

The mean(s.d.) duration of surgery was 210(42) min, and that for the perineal stage was 72(15) min. In later stages of enrolment, six patients were operated on with double teams, with a mean(s.d.) duration of surgery of 150(32) min (*P* = 0·002).

Postoperative outcomes for the two groups are shown in *Table* 2. One patient with mid rectal cancer reported difficulty in voiding (grade II), but this resolved after 1 month with conservative treatment. Three presacral abscesses were documented by clinical signs (fever, anal pain), clinical examination, abdominal–pelvic CT/pelvic MRI, and considered as major complications (grade III). All patients were treated with a surgical procedure that avoided leakage: transanastomotic drainage was performed (4 o'clock position) using a Kelly forceps, to place a tube. The drain was removed on day 3 after surgery. Drainage openings were closed 2 weeks later, and closure of the ileostomy was performed after 4 weeks in all patients. Continence was classified as grade I in all patients at 3 months after ileostomy closure.

**Table 2:** Postoperative outcomes

	Mid rectal cancer (*n* = 25)	Low rectal cancer (*n* = 13)	*P* ‡
**Duration of surgery (min)** *	217(22)	204(31)	0·143§
**Duration of perineal operation (min)** *	75(17)	67(12)	0·140§
**Length of postoperative stay (days)** *	7·1(2·3)	6·9(2·6)	0·809§
**Clavien–Dindo complication grade**			
0	0	0	
I	0	0	
II	1	0	0·473
III	1	2	0·220
IV	0	0	
Overall	2	2	0·481
**(y)pT status**			
(y)pT1	0	0	
(y)pT2	15	5	0·215
(y)pT3	9	5	0·880
(y)pT4	1	3	0·071
**(y)pN status**			
(y)pN0	18	9	0·852
(y)pN1	7	4	0·864
(y)pN2	0	0	
**(y)pCRM**			0·034
Positive	0	3	
Negative	25	10	
**TME grade**			0·038
1	24	9	
2	1	4	
3	0	0	
**(y)pDRM**			
Positive	0	0	
Negative	25	13	
**Sphincter function grade at 3 months**			
I	25	13	
II	0	0	
**Recurrence**	2	0	0·301
Local	1†	0	0·472
Distal	1	0	0·472

* Values are mean(s.d.).

† Patient died at 18 months. CRM, circumferential resection margin; TME, total mesorectal excision; DRM, distal resection margin.

‡ Fisher's exact test, except

§ independent *t* test.

Low rectal tumours had a significant rate of CRM positivity on pathological examination compared with mid rectal tumours (*P* = 0·034) and significantly more patients in this group had TME grade 2 (*P* = 0·038), although these tumours did have a significantly larger diameter (*P* = 0·035). Patients with CRM positivity or TME grade 2 were referred for adjuvant chemotherapy; no significant differences in oncological outcomes were documented.

After a median follow‐up of 17 months, one woman with mid rectal cancer (T3 N1 status, CRM negativity, TME grade 2 macroscopic assessment) died from local recurrence that invaded the urinary bladder and left ureter; this was documented by colonoscopy and abdominal–pelvic CT. One man with mid rectal cancer (T3 N1 status) had liver metastasis at 6 months (*Table* 2).

**Figure 2:**
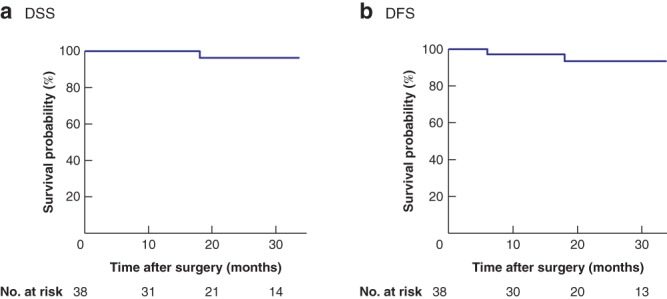
Kaplan–Meier analysis of disease‐specific and disease‐free survival in patients with rectal cancer **a** Disease‐specific survival (DSS); **b** disease‐free survival (DFS).

For the whole cohort, DSS and DFS were 41·0 and 40·2 months respectively (*Fig*. 2).

## Discussion

Laparoscopic TME for mid–low locally advanced rectal cancer is a difficult procedure, especially in men with a narrow pelvis or increased BMI^1^, ^2^, ^3^, ^4^, ^5^. Moreover, in patients who undergo preoperative chemoradiotherapy, difficulty in identifying dissection planes^20^, ^21^, ^22^, ^23^, ^24^ could lead to the conversion rate ranging from 1·2 to 28 per cent^22^.

TaTME has the advantage of combining minimally invasive treatment with a standard oncological approach. A possible concern involves the transanal extraction of bulky tumours. For this reason, and to avoid anal sphincter lesions, specimens with tumour more than 5 cm in diameter were extracted via an abdominal incision in the right lower quadrant (planned for protective ileostomy), as performed by others^25^, ^26^, ^27^, ^28^, ^29^.

Reports documenting higher rates of positive pelvic culture and presacral abscess were also of concern^10^, although a literature review showed significantly lower rates^9^, ^11^, ^12^. The number of presacral abscesses reported in the present study (3 of 38) exceeded these previously reported values; this could relate to the long time for which the rectal stump was in the pelvis at the start of the study. Consistent with this hypothesis, when a double‐team approach was used in this series and the rectal stump was in the pelvis for a shorter time, no presacral abscesses developed.

A recent comprehensive systematic review^11^ of 33 studies including 661 patients who had TaTME showed TME to be 87·6 per cent complete, 10·9 per cent nearly complete and 1·5 per cent incomplete. The authors reported a 0·2 per cent rate for DRM positivity and 4·7 per cent for CRM positivity. Rates of CRM positivity in the present study were significantly higher in patients with low rectal cancer than in those with mid rectal cancer, although the sample was very small and low rectal cancers had an increased mean diameter.

A factor that may influence sphincter function particularly after TaTME is the prolonged anal dilatation owing to the wide anal platforms that are involved throughout the procedure. Rates of severe low anterior resection syndrome following TaTME vary significantly in the literature, ranging from 10 to 82 per cent, although results were from very small cohorts^30^, ^31^, ^32^.

Recently, the Norwegian Radium Hospital in Oslo reported an unexpected pattern of recurrences occurring early after TaTME; ten local recurrences (9·5 per cent) were diagnosed a median of 11 months after surgery^13^. However, the local recurrence rate at a median of 17 months in the present study (1 of 38, 2·6 per cent) was comparable with that in several systemic reviews^11^, ^33^, ^34^.

TaTME offers a combined laparoscopic and transanal approach to achieve a safe and oncologically complete TME dissection for locally advanced mid–low rectal tumours. However, bulky low rectal tumours require special attention to reduce the rate of CRM positivity.

## Disclosure

The authors declare no conflict of interest.
